# Comparative Transcriptome Analysis Identifies Putative Genes Involved in Dioscin Biosynthesis in *Dioscorea zingiberensis*

**DOI:** 10.3390/molecules23020454

**Published:** 2018-02-18

**Authors:** Jia Li, Qin Liang, Changfu Li, Mengdi Liu, Yansheng Zhang

**Affiliations:** 1 CAS Key Laboratory of Plant Germplasm Enhancement and Specialty Agriculture, Wuhan Botanical Garden, Chinese Academy of Sciences, Wuhan 430074, China; lijia@wbgcas.cn (L.J.); liangqin2017@163.com (Q.L.); lichangfu@wbgcas.cn (C.L.); liumengdi15@mails.ucas.edu.cn (M.L.); 2 College of Life Sciences, University of Chinese Academy of Sciences, Beijing 100049, China

**Keywords:** *Dioscoreazingiberensis*, dioscin biosynthesis, transcriptome, cytochrome P450, glycosyltransferase

## Abstract

*Dioscorea zingiberensis* is a perennial herb native to China. The rhizome of *D. zingiberensis* has long been used as a traditional Chinese medicine to treat rheumatic arthritis. Dioscin is the major bioactive ingredient conferring the medicinal property described in Chinese pharmacopoeia. Several previous studies have suggested cholesterol as the intermediate to the biosynthesis of dioscin, however, the biosynthetic steps to dioscin after cholesterol remain unknown. In this study, a comprehensive *D. zingiberensis* transcriptome derived from its leaf and rhizome was constructed. Based on the annotation using various public databases, all possible enzymes in the biosynthetic steps to cholesterol were identified. In the late steps beyond cholesterol, cholesterol undergoes site-specific oxidation by cytochrome P450s (CYPs) and glycosylation by UDP-glycosyltransferases (UGTs) to yield dioscin. From the *D. zingiberensis* transcriptome, a total of 485 unigenes were annotated as CYPs and 195 unigenes with a sequence length above 1000 bp were annotated as UGTs. Transcriptomic comparison revealed 165 CYP annotated unigenes correlating to dioscin biosynthesis in the plant. Further phylogenetic analysis suggested that among those CYP candidates four of them would be the most likely candidates involved in the biosynthetic steps from cholesterol to dioscin. Additionally, from the UGT annotated unigenes, six of them were annotated as 3-*O*-UGTs and two of them were annotated as rhamnosyltransferases, which consisted of potential UGT candidates involved in dioscin biosynthesis. To further explore the function of the UGT candidates, two 3-*O*-UGT candidates, named Dz3GT1 and Dz3GT2, were cloned and functionally characterized. Both Dz3GT1 and Dz3GT2 were able to catalyze a C3-glucosylation activity on diosgenin. In conclusion, this study will facilitate our understanding of dioscin biosynthesis pathway and provides a basis for further mining the genes involved in dioscin biosynthesis.

## 1. Introduction

*Dioscorea zingiberensis*, commonly known as “yellow ginger” in China, is extensively cultivated as a traditional medicinal crop in the southern region of Shaanxi province in China. Extracts from *D. zingiberensis* rhizomes have been suggested in Chinese medicine for the treatment of chest pain, coronary heart disease, and hypoglycemic problems [1]. These medicinal effects are mostly attributed to the presence of steroidal saponins in its rhizome tissue [2], and of which dioscin is the principle active component [3]. A variety of bioactivities, such as anti-tumor [4], anti-inflammatory [5], and bone-protecting [6] properties, have been shown for dioscin. More importantly, the aglycone of dioscin, namely diosgenin, serves as the most important starting material for the synthesis of many types of sterol drugs, such as progesterone, androgen, and contraceptives [7]. 

**Figure 1:**
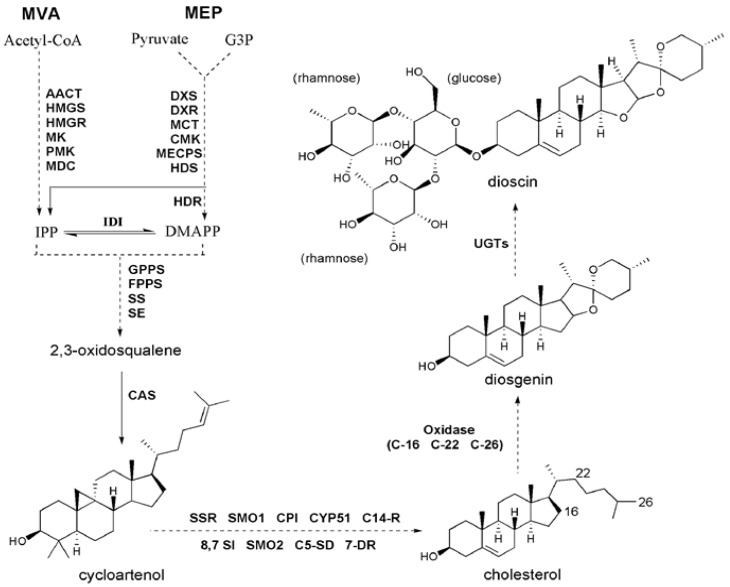
Putative pathway for dioscin biosynthesis in *D. zingiberensis*. Abbreviations used are: AACT, acetyl CoA acetyltransferase; HMGS, hydroxymethyl glutaryl CoA synthase; HMGR, 3-hydroxy-3-methylglutaryl CoA reductase; MK, mevalonate kinase; PMK, phosphomevalonate kinase; MDC, mevalonate diphosphosphate decarboxylase; IPI, IPP diphosphateisomerase; DXS, 1-deoxy-D-xylulose-5-phosphate synthase; DXR, 1-deoxy-D-xylulose-5-phosphate reductoisomerase; MCT, 2-C-methyl-D-erythritol 4-phosphate cytidylyl transferase; CMK, 4-diphospho-cytidyl-2-C-methyl-D-erythritol kinase; MECPS, 2-*C*-methyl-D-erythritol-2, 4-cyclodiphosphate synthase; HDS, 4-hydroxy-3-methylbut-2-(*E*)-enyl diphosphate synthase; HDR, 4-hydroxy-3-methylbut-2-enyldiphosphate reductase; GPPS, geranyl diphosphate synthase; FPPS, farnesyl diphosphate synthase; SS, squalene synthase; SE, squalene epoxidase; CAS, cycloartenol synthase; SSR, sterol side chain reductase; SMO1, sterol 4a–methyl oxidase 1; SMO2, sterol 4a-methyl oxidase 2; CPI, cycloeucalenol cycloisomerase; CYP51, sterol C-14 demethylase; C14-R, sterol C-14 reductase; 8,7-SI, sterol 8,7-isomerase; C5-SD, sterol C-5(6) desaturase; 7-DR, 7-dihydrocholesterol reductase.

Despite of the medicinal importance of dioscin, there is not yet a complete list of genes identified for its biosynthesis, especially the genes coding for tailoring enzymes in the late steps to dioscin biosynthesis are missing at present. Previous labeling studies have suggested cholesterol as the precursor for dioscin biosynthesis [8,9,10]. In plants, cholesterol is synthesized via the isoprenoid biosynthetic pathway through 2,3-oxidosqualene followed by cycloartenol synthase, and genes involved in the steps from cycloartenol to cholesterol have been discovered (Figure 1) [11]. Cholesterol can be transformed into dioscin through modifications which include oxidation at the C-22, C-26, and C-16 positions by enzymes such as P450-dependent monooxygenases, dioxygenases, and other catalysts, and subsequent addition of glucose and rhamnose groups to the C-3 position of diosgenin by UDP- glycosyltransferases (UGTs) (Figure 1). Although the specific steps in dioscin biosynthesis starting from cholesterol were previously proposed by Mehrafarin et al [12], the proposed steps have never been experimentally validated, and genes involved in these steps have never been isolated.

**Figure 2:**
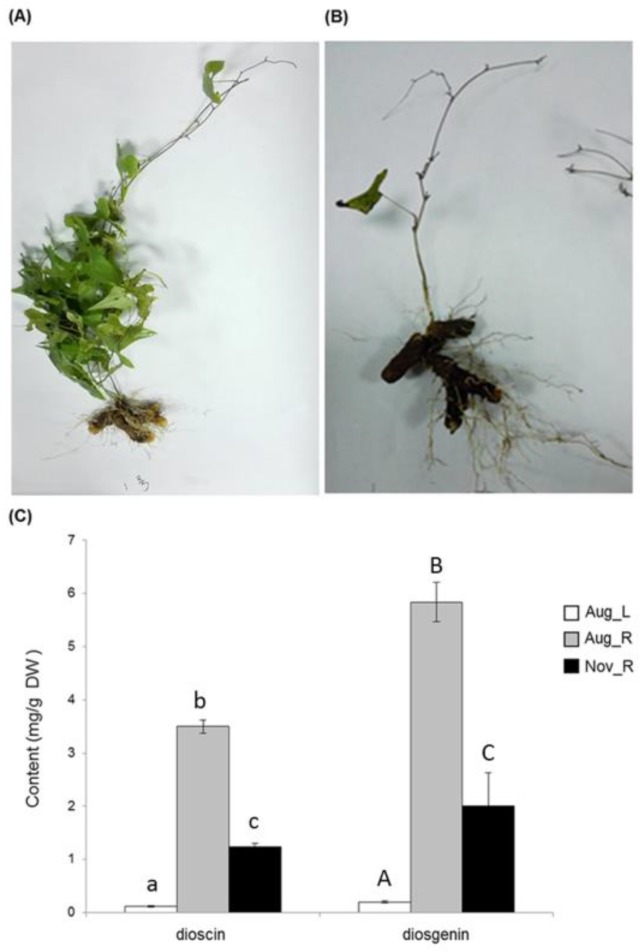
Phytochemical analysis of the leaf and rhizome tissues of *D. zingiberensis*. (**A**,**B**) representative pictures are shown for the *D. zingiberensis* plants collected in August (**A**) and November (**B**) of 2015; (**C**), the content of diosgenin and dioscin in the different plant parts, including the leaves harvested in August (Aug_L), the rhizomes harvested in August (Aug_R), and the rhizomes harvested in November (Nov_R). Two biological replicates were included for this experiment. Different letters on the bar indicate a significant difference at *p* < 0.05 based on generalized linear models with Bonferroni multicomparison tests.

Advances in RNA sequencing technology provide an efficient and economical platform for gene discovery in non-model plants without knowing their genome sequences [13]. Owing to the tissue-specificity for the synthesis of many types of plant metabolites, transcript profiling together with metabolite analysis from different tissues could prove valuable for the identification of genes in different metabolic pathways [14,15,16]. We have confirmed that dioscin is highly synthesized in the underground rhizome tissue of *D. zingiberensis* while in the aerial leaf tissue it is formed at very low concentrations (Figure 2). Thus, to identify specific genes putatively in the downstream steps to dioscin biosynthesis, transcriptomic comparison between these two tissues was conducted and extensively analyzed in this study. Genes encoding several members of cytochrome P450 family and glycosyltransferase group, which could be putatively involved in the late steps to dioscin biosynthesis, have been identified. By heterologous expression in *E. coli*, two UGTs capable of glucosylating diosgenin on C-3 position were functionally confirmed.

## 2. Results and Discussion

### 2.1. Generation of D. Zingiberensis Transcriptomes from its Leaf and Rhizome Tissues

It is well known that rhizome tissue is the richest source for dioscin production in *D. zingiberensis*. We confirmed this tissue-specificity for dioscin as well as its aglycone diosgenin by measuring their contents in its leaf and newly-formed rhizome tissues, which we collected at a relatively earlier stage on 28 August 2015 (Figure 2A). Rhizomes (five months old) were shown to produce higher amounts of dioscin and diosgenin (3.50 mg g^−1^ for dioscin and 5.84 mg g^−1^ for diosgenin) while in leaf tissue (five months old) much less of them were detected (0.12 mg g^−1^ for dioscin and 0.20 mg g^−1^ for diosgenin) (Figure 2C). At the later stage (2 November 2015), most of leaves faded and fell off from the plants (Figure 2B), and in the rhizome tissue (eight months old) of this stage the biosynthesis of either dioscin or diosgenin largely decreased in comparison with that in the younger rhizomes (1.23 mg g^−1^ of dioscin and 2.00 mg g^−1^ of diosgenin were detected from the rhizomes of this later stage) (Figure 2C). 

Based on the metabolite accumulation pattern described above, the leaf (described as Aug_L) and newly-formed rhizomes (Aug_R) harvested at the younger stage, and the newly-formed rhizomes (Nov_R) harvested at the later stage were chosen for comparative transcriptome analysis to understand the molecular mechanism underlying dioscin biosynthesis. Total RNA from each tissue sample was prepared and was sent to Novogene Company (Beijing, China) for the cDNA library construction and the RNA-Seq analysis. The number of the resulting raw reads, clean reads, Q20, Q30, GC content of each sample, and N50 is shown in Table 1. All the raw data have been submitted to the NCBI sequence read archive (SRA; http://www.ncbi.nlm.nih.gov/sra) under the accession number of SRR6281651 for Aug_L, SRR6281650 for Aug_R, and SRR6281649 for Nov_R, respectively. The *de novo* assembly of all the clean reads was performed using Trinity software. A total of 176,406 transcripts were assembled with a mean length of 715 bp in a range of 201 to 16,773 bp. Transcripts were further assembled into 143,245 unigenes and the assembled unigenes were then searched against several public databases including NCBI non-redundant protein database (Nr), NCBI nucleotide sequences database (Nt), Swiss-Prot protein database, Pfam database, Clusters of Orthologous Groups of protein databases (COG) and Gene ontology (GO). In total, 74,908 (52.29%) unigenes were successfully annotated in at least one of the above public databases (Table S1). According to Nr annotation, 8229 (16.2%) unigenes had the most hits from *Elaeis guineensis*, followed by *Phoenix dactylifera* (6857, 13.5%), *Musa acuminate* (2692, 5.3%), *Hordeum vulgare* (2387, 4.7%), and *Vitis vinifera* (1117, 2.2%). 

### 2.2. CD-HIT and BlastP Analysis

Unigene sequences were clustered by Cluster Database at High Identity with Tolerance (cd-hit) to reduce the redundancy. The 143,245 uigenes were divided into 143,095 clusters. The result was shown in Table S2. After doing the blast, the amino acid sequences of 407 CYP unigenes have some homology (from 21.15% to 86.55%) to the CYP450 proteins from *Arabidopsis thaliana*, while the amino acid sequences of 488 UGT unigenes show different sequence identities (from 19.57% to 83.33%) to the UGT proteins from *Arabidopsis thaliana*. The results were shown in Table S3 and Table S4, respectively.

### 2.3. Identification of Genes Related to Steroidal Backbone Biosynthesis

Steroidal saponins are biosynthesized from C5 units, isopentenyl diphosphate (IPP), which is derived either from the cytosolic mevalonate pathway (MVA pathway) or from the plastidic methylerythritol phosphate pathway (MEP pathway). In the *D. zingiberensis* transcriptome of this study, eleven unigenes putatively code for the MVA pathway enzymes (Table 2), including three for AACT, one for HMGS, one for HMGR, two for MK, one for PMK, one for MVD, and two for IDI; seven unigenes were identified as the putative genes in the MEP pathway (Table 2), which included two for DXS, one for DXR, one for CMK, one for MECPS, one for HDS and one for HDR. Among the MVA pathway unigenes, the transcripts of the c46893_g1 and c102447_g1 coding for AACT, the c76083_g1 for MK, the c61824_g1 for HMGS, and the c74391_g1 for IDI were specifically expressed in the rhizomes (Aug_R) but not in the leaves (Aug_L), which is in accordance with the rhizome-specificity for dioscin biosynthesis in *D. zingiberensis*. On the other hand, all the MEP pathway backbone unigenes were expressed at much higher levels in the leaves (Aug_L) than in the rhizomes (Aug_R), and their expressions in both rhizome tissues (Aug_R and Nov_R) were essentially similar (Table 2), which does not match the metabolite accumulation pattern (Figure 2C). These results indicate that dioscin biosynthesis is likely to be mainly from the MVA pathway. 

In the *Solanaceae* plant family, especially those producing the steroidal glycoalkaloids (SGAs), there are two separate sets of pathway enzymes responsible for the biosynthesis of cholesterol and phytosterols [11]. However, based on the bioinformatics analysis here, we proposed the same set of enzymes probably occurring in the cholesterol and phytosterol pathways in *D. zingiberensis*. A similar case was also previously suggested for another diosgenin-producing plant *Trigonella foenum-graecum* [17]. The unigenes encoding all the known pathway enzymes from IPP to cholesterol were discovered in the *D. zingiberensis* transcriptome (Table 2), including two for GPPS, one for FPPS, one for SS, one for SE, one for CAS, two for SSR, one for SMO1, one for CYP51, one for C14-R, one for 8,7 SI, one for SMO2, one for C5-SD, and one for 7-DR. Except for the unigenes putatively coding for GPPS, FPPS, CAS, CPI, and 8,7 SI, all the other unigenes in the pathway were more highly expressed in the rhizomes (Aug_R) than in the leaves (Aug_L) and their transcript abundances were much higher in the younger rhizomes (Aug_R) than in the mature rhizomes (Nov_R) (Table 2). Taken together, except for the MEP pathway genes displaying higher expression in the leaves (Aug_L), most of the unigenes involved in the steroidal saponin backbone biosynthesis showed higher expression in the Aug_R, followed by the Nov_R, and Aug_L(Table 2), which parallels to the accumulation pattern of diosgenin or dioscin in the plants (Figure 2C). This data implicates that the biosynthesis of the steroidal metabolites in *D. zingiberensis* is highly controlled by the transcriptional regulation of steroidal backbone biosynthesis genes.

### 2.4. Identification of CYP450 Unigenes Putatively Involved in Diosgenin Biosynthesis

It has been suggested that diosgenin is biosynthesized from the steroidal skeleton compound cholesterol by a series of oxidative reactions at the C-22, C-26, and C-16 positions [18] (Figure 1). The hydroxylation of cholesterol is likely catalyzed by cytochrome P450 (CYP) enzymes. Through annotation using different databases, a total of 485 CYP-encoding unigenes were discovered in *D. zingiberensis* transcriptome (Table S5), among which 165 CYP unigenes showed the highest expression level in Aug_R, followed by Nov_R and Aug_L (Table S6), coincident with the accumulation pattern of diosgenin in those tissue samples. These CYP unigenes exhibiting correlating expression pattern with diosgenin accumulation were considered as the potential candidates to be involved in diosgenin biosynthesis. Among them, there were 22 CYP unigenes putatively being presented at full-length cDNAs (Table S6), out of which the unigene c56203_g1 was annotated as a sterol 14α- demethylase. It is well known that the sterol 14α-demethylase functions in the pathway steps to cholesterol formation [11]. Cholesterol was proposed to be the precursor for diosgeninbiosynthesis, and thus identification of sterol 14α- demethylase annotated unigene within our list of the potential P450 candidates indicates that some of the other CYP candidates might play an important role in diosgenin biosynthesis. We performed phylogenetic analysis for these full-length CYP candidates together with the well characterized CYPs from various metabolic pathways, including the ones that are relevant to the biosynthesis of triterpenoids and steroidal saponins. The 2-oxoglutarate-dependent dioxygenases from potato (St16DOX) and tomato (Sl16DOX), which oxidize hydroxycholesterols at the C-16 position [19], were included in the alignments as the outgroup.

**Figure 3:**
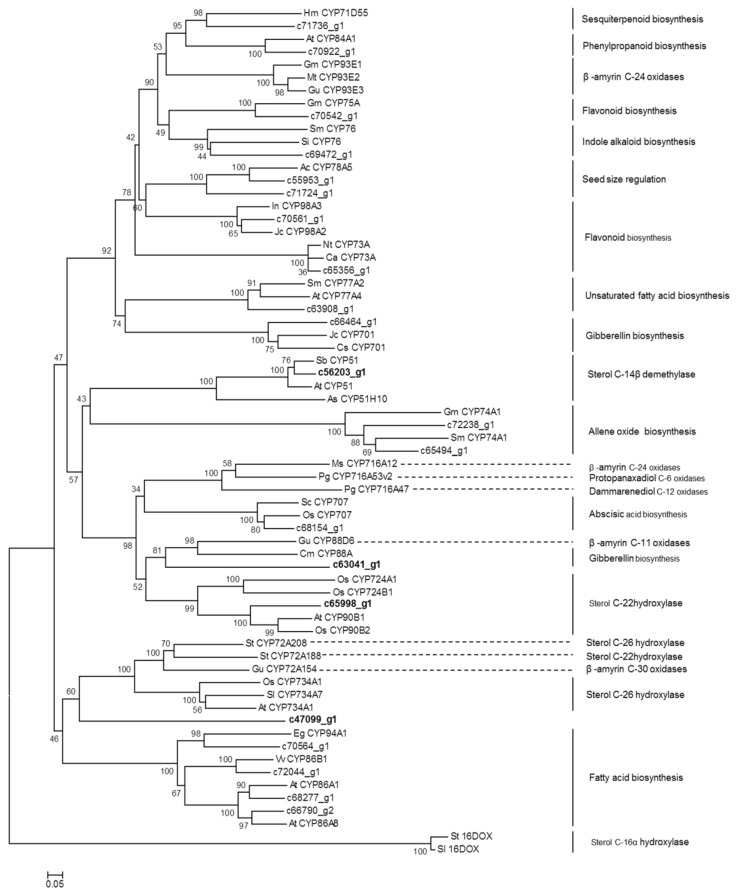
Phylogenetic analysis of potential CYP450 candidates from *D. zingiberensis* transcriptome with previously published CYP450s from various metabolic pathways.

As shown in Figure 3, the unigene c65998_g1, which belongs to the CYP90B subfamily, grouped together with AtCYP90B1 from *A. thaliana* [20], and OSCYP90B2 and OsCYP724B1 from rice [21] that were all characterized to be a steroid C-22 hydroxylase in brassinosteroid pathway, strongly suggesting that the unigene c65998_g1 might be the CYP candidate responsible for the cholesterol C-22 oxidation in diosgenin biosynthesis. It should be noted that a CYP enzyme StCYP72A188 from potato, which also catalyzes a sterol C-22 hydroxylation in steroidal glycoalkaloid (SGA) biosynthesis [22], was in the clade distinctly different from the above mentioned sterol C-22 hydroxylases (Figure 3). Also, among the full-length CYP candidates matching the accumulation of diosgenin (Table S6), no members of the CYP72A group were found. These data suggest that the C-22 hydroxylase in SGA pathway might evolve independently from the CYPs for diosgenin or brassinosteroid pathway. The unigene c47099_g1, which belongs to CYP505 family, showed a relatively closer relationship to the sterol C-26 hydroxylases that include SlCYP734A7 from *Solanum lycopersicum* [23], OsCYP734A1 from rice [24] (Sakamoto et al., 2011) , and StCYP72A208 from potato [22], suggesting that the unigene c47099_g1 might encode the C-26 hydroxylase in diosgenin biosynthesis. 

CYP enzymes displaying the sterol C-16 hydroxylation activity have not been identified yet, however, a 2-oxoglutarate-dependent dioxygenase (designated St16DOX) has recently been reported to play a role in SGA biosynthesis in potato by catalyzing the C-16 oxidation on hydroxycholesterols [19]. Its equivalent in tomato, designated Sl16DOX, has essentially the same biochemical activity as does St16DOX [19]. From the *D. zingiberensis* transcriptome data, we searched for close homologs of St16DOX. However, the closest homolog unigene c51094_g1 displayed only 36% amino acid identity while showing a 77% identity to Oryza sativa *Japonica* 2-oxoglutarate-Fe-dioxygenase. The unigene c51094_g1 was specifically found in the *D. zingiberensis* leaf tissue whereas was not observed in the rhizome (data not shown). It still could not be ruled out that there exist CYP enzymes catalyzing the sterol C-16 oxidation for diosgenin biosynthesis. Phylogenetic analysis of the unigene c63041_g1 revealed a relatively closer relationship to genes encoding the sterol C-22 hydroxylases and a β-amyrin (a triterpenoid) C11-oxidase [25]. The c63041_g1 shows similarity to genes encoding enzymes belonging to CYP88D group, and from this family some members have already been reported to participate in the biosynthesis of SGA [26] or triterpenoids [25]. Thus, it will be a priority to test whether the unigene c63041_g1 participates in diosgenin biosynthesis as a C-16 oxidase. The rest of the CYP unigenes were grouped into many other P450 subfamilies (i.e., CYP76, CYP75A, CYP71D55, CYP93E, CYP78A5, CYP73A, CYP77A, CYP701, CYP74A, CYP716A, and CYP707), which are known to participate in several other pathways, including indole alkaloid, flavonoid, sesquiterpenoid, fatty acid, gibberellin, and Abscisic acid (ABA) metabolisms (Figure 3).

### 2.5. Identification of UGT Candidates Involved in Dioscin Biosynthesis 

The biosynthesis of dioscin requires the addition of glucose and rhamnosegroups onto the C-3 hydroxyl group, which is catalyzed by UGTs (Figure 1). In the present study, 195 unigenes encoding UGTs with a sequence length above 1000 bps were obtained (Table S4). Inspection of these UGT unigenes revealed six unigenes (c73651_g1, c70118_g1, c61522_g1, c66977_g1, c60958_g1, and c59129_g1) annotated as a sterol 3-*O*-UGT and two unigenes (c60525_g2 and c69762_g2) as a UDP-rhamnose: rhamnosyltransferase (Table S7). These UGT unigenes would be possible candidates for dioscin biosynthesis. However, the expression trend of all the identified UGTs (Table S7) did not match the distribution pattern of dioscin in the tissue samples (Figure 2C). A similar case also has been reported for *Panax japonicas* UGTs whose expression pattern across various tissues is different from the tissue-specificity for triterpenoid saponin biosynthesis in *Panax japonicas* [27]. These data indicate that glycosylation reaction governed by UGTs might not influence the tissue-specificity of saponin synthesis in some of the plant species. 

**Figure 4:**
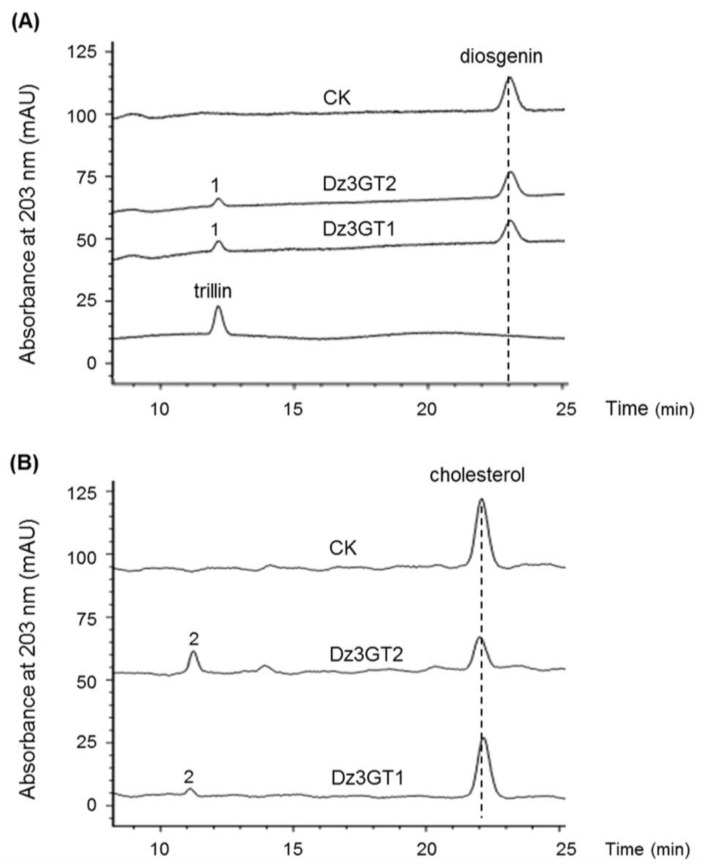
In vitro C3-glycosylation of diosgenin and cholesterol by the purified recombinant Dz3GT1 and Dz3GT2. HPLC profiles were shown for the assays with diosgenin (**A**) and cholesterol (**B**). CK means the control reaction. For the controls, the purified Dz3GTs were excluded from the reaction mixtures. Both Dz3GT1 and Dz3GT2 were able to convert diosgenin to one new product (peak 1), corresponding to trillin (diosgenin 3-*O*-glucoside), which was not produced in the controls. When cholesterol was used as the substrate, a new product (peak2) was produced by both the UDP-glycosyltransferase (UGT) proteins but was not observed from the control assays. The peak 2 was assumed to be cholesterol 3-*O*-glucoside based on the C3-glucosylating activities of Dz3GTs.

For biochemical characterizations of the identified UGTs, it is necessary to know their full open reading frames (ORFs). Unfortunately, only partial transcripts of the two rhamnosyltransferase candidates were present in the transcriptome. Among the identified sterol 3-*O*-UGTs, the unigenes of c60958_g1 and c59129_g1 were expressed at very low levels in all the tissue samples, moreover, they were not at a full-length and thus were not subjected to further analysis. Within the rest of the 3-*O*-UGTs, only the c61522_g1 and c66977_g1 contain complete ORFS. The gene products of c61522_g1 and c66977_g1 were designated as Dz3GT1 (NCBI accession no. MG488289) and Dz3GT2 (NCBI accession no. MG488290), respectively, in this study. Dz3GT2 shows only 56% amino acid identity to Dz3GT1 but displays 99.8 % identity to the previously identified DzS3GT from *D. zingiberensis,* the enzyme catalyzing a C3-glucosylating activity on diosgenin [28]. To further characterize the biochemical properties of Dz3GT1 and Dz3GT2, their ORFs were transferred into *E. coli* cells, and the recombinant UGTs were purified by the use of their N-terminal fused tags. The recombinant protein was assayed with diosgenin and cholesterol followed by HPLC analysis. Both Dz3GT1 and Dz3GT2 were able to convert diosgenin to a new product (peak 1) which corresponds to trillin (diosgenin 3-*O*-glucoside) authentic standard (Figure 4A), suggesting their 3-*O*-glucosylating activities on diosgenin. When cholesterol was used as a substrate, a new product (peak 2) was observed from the assays with both *D. zingiberensis* UGTs (Figure 4B). Based on the C3-glucosylating activities of Dz3GTs described above, we assumed the peak 2 to be cholesterol 3-*O*-glucoside, although the cholesterol 3-*O*-glucoside standard is not commercially available at present. Both Dz3GT1 and Dz3GT2 function redundantly in C3-*O*-glucosylation of diosgenin, the importance of Dz3GT1 relative to Dz3GT2 in dioscin formation is not clear. Further characterization of Dz3GT1 and Dz3GT2 is required to confirm their roles in dioscin formation by silencing their gene expression in *D. zingiberensis*.

## 3. Materials and Methods 

### 3.1. Plant Material

Wild *D. zingiberensis* plants were collected from Zhuxi County, Hubei province of China. On 12 March 2015, healthy rhizomes were planted in a field managed by the Wuhan Botanical Garden of Chinese Academy of Science. After being grown for about five months, samples of the newly-formed rhizome and young leaf tissues were harvested on 28 August 2015 to identify genes differentially transcribed in both tissues. To monitor the gene expression change in mature (eight month old) rhizomes relative to the younger (five month old) ones, the newly-formed rhizomes at a late developmental stage were also harvested on 2 November 2015. It should be noted that we did not collect leaf tissue at this later stage because at that age most of the leaves faded and fell off from the plants. All the harvested plant materials were flash frozen in liquid nitrogen after cleaning and stored at −80°C until use.

### 3.2. Transcriptome Sequencing, De Novo Assembly and Unigene Functional Annotation

Total RNA were isolated from the rhizome and leaf tissues using a plant RNA Prep Kit (Tiangen Biotech, Beijing, China) following the product manual. Each tissue was sampled from two individual plants, and after quality determination RNA from two biological repeats was mixed in equal amounts into a single pool for RNA-sequencing. The RNA samples were sent to Novogene Company (Beijing, China) where the cDNA libraries were constructed and RNA-sequencing was performed on an Illumina HiSeq2000 platform. Raw reads were cleaned by removing adapter containing reads and low quality reads. To monitor sequencing quality, the values of Q20 and Q30 of the clean data were determined. Clean reads were *de novo* assembled by Trinity software with default parameters [29]. Gene function was annotated by BLASTx (*E*-value < 1e^−5^) search against NR, Nt KOG/COG, Swiss-Prot, KO (KEGG Ortholog), Pfam, and GO databases. To calculate gene expression levels, clean reads were mapped back to the assembled transcriptome to get read-counts for each unigene and gene expression levels were normalized to Fragments Per kb per Million fragments (FPKM) [30].

### 3.3. CD-HIT Analysis

Cd-hit-est was used to cluster the Unigene sequences to reduce the redundancy (http://weizhongli-lab.org/cdhit_suite/cgi-bin/index.cgi). Sequence identity cut-off was set as 0.9.

### 3.4. Blastp Analysis

The amino acid sequences of CYP unigenes from the *D. zingiberensis* transcriptome data were compared to the CYP450 protein database from *Arabidopsis thaliana (*http://drnelson.uthsc.edu/P450seqs.dbs.html), while the amino acid sequences of UGT unigenes were compared to the UGT protein database from *Arabidopsis thaliana* (http://www.p450.kvl.dk/UGT.shtml) by BLASTP 2.2.31+.

### 3.5. Phylogenetic Analysis

The open reading frame of identified CYP450s was predicted by using Translate tool (http://www.expasy.ch/tools/dna.html/). Amino acid sequence alignment was performed using ClustalW program (http://www.ebi.ac.uk/clustalW/). A phylogenetic tree was constructed by neighbor-joining method using MEGA 5 software [31].

### 3.6. Plant Metabolite Extraction

To measure diosgenin content, plant tissue was ground into fine powder in liquid nitrogen and extracted with methanol under sonication (180 W, 40 kHz, 30 °C, 20 min). The methanol extracts were dried in a vacuum concentrator and hydrolyzed with 1.5 M sulfuric acid for 4 h at 100 °C. After acid hydrolysis, the residue was extracted with petroleum ether and washed to neutral. The petroleum ether extracts were evaporated to dryness and re-dissolved in methanol prior to HPLC analysis. To measure dioscin content, powdered tissue was extracted with methanol, and the methanol extracts were dried in a vacuum concentrator and re-dissolved in methanol for HPLC analysis.

### 3.7. Recombinant Protein Expression in E. Coli and Purification

The full length *Dz3GT2* was amplified and sub-cloned into the pET28a vector (Novagen, Darmstadt, Germany) to give an in-frame N-terminal fusion with a His tag. The *Dz3GT2* expression vector was then transformed into BL21 (DE3) *E. coli* competent cells. *Dz3GT1* was initially cloned into the pET28a vector with a fusion with a His tag, but this construct gave the recombinant Dz3GT1 only expressed in inclusion bodies but not in a soluble form. To get a soluble Dz3GT1, the full length *Dz3GT1* was then sub-cloned into the pGEX-2T vector which encodes an N-terminal GST-tagged fusion protein of Dz3GT1. The *Dz3GT1* expression vector was transformed into BL21 *E. coli* competent cells. The transformed *E. coli* cells were grown in Luria–Bertani (LB) medium and the protein expression was induced with the addition of 1 mM isopropyl-1-thio-b-D-galactopyranoside (IPTG) into the medium. Following the manufacturer’s protocol, the soluble recombinant Dz3GT2 was purified using Nickel affinity HisTrap™ column (GE Healthcare) while Dz3GT1 was purified by the use of a glutathione sepharose 4B kit (GE Healthcare).

### 3.8. Enzyme Assays

The activity of the recombinant UGT was tested in a 200 µL reaction mixture containing 50 mM Tris-HCl buffer (pH 8.0), 200 µM substrate (sugar acceptor), 2 mM dithiothreitol (DTT), 2 mM UDP-glucose and 3 μg of the purified UGT. Controls were performed by omitting the purified UGT in the reaction mixture. After incubating the reaction mixture overnight at 30 °C, the reaction was stopped with 200 µL methanol and the reaction products were directly subjected to HPLC analysis.

### 3.9. HPLC (High Performance Liquid Chromatography) Analysis

HPLC analysis was performed on an LC-20AT instrument (Shimadzu, Kyoto, Japan) using an inertsil ODS-SP reverse phase column (250 mm × 4.6 mm, 5 µm) at 30 °C. The monitoring wave length was set to 203 nm. Milli-Q water (solvent A) and HPLC-grade methanol (solvent B) were used as the mobile phase. To measure diosgenin content in the plant materials or analyze the products from the in vitro assays with diosgenin, the samples were separated using 90% B for 30 min at a flow rate of 1 mL/min. For detecting the products from the enzyme assays with cholesterol, the samples were eluted in 98% B. For measuring diosin content in the plant materials, the separation was achieved with milli-Q water (solvent A) and HPLC-grade acetonitrile (solvent B) as the mobile phase at a flow rate of 0.8 mL/min, and the solvent gradient was set as follows: 0–20 min, 10–90% B; 20–30 min, 90–10% B; 30–40 min, 10% B.

## Figures and Tables

**Table 1:** Summary of the sequencing data.

Item	Sample	Number	Q20(%)	Q30(%)	GC(%)	N50(bp)
Raw read	Aug_L	55,798,190	ND	ND	ND	ND
	Aug_R	45,367,360	ND	ND	ND	ND
	Nov_R	42,564,054	ND	ND	ND	ND
Clean read	Aug_L	53,330,426	97.68	94.42	49.52	ND
	Aug_R	43,234,636	97.68	94.44	47.54	ND
	Nov_R	40,686,226	97.72	94.51	47.87	ND
Total	Unigenes	143,245	ND	ND	ND	814

ND: no data.

**Table 2:** Transcriptomic comparison of putative cholesterol pathway genes across different tissues of *D. zingiberensis.*

	Enzyme Name	Gene ID	FPKM (Aug_R)	FPKM (Aug_L)	FPKM (Nov_R)	log2ratio (Nov_RvsAug_R)	q-Value (Nov_RvsAug_R)	log2ratio (Aug_LvsAug_R)	q-Value (Aug_LvsAug_R)
MVA									
	AACT-1	c46893_g1	4.86	0	0.17	−5.1594	0.13758	−3.6187	0.18281
	AACT-2	c72984_g1	38.7	32.07	67.57	0.59302	1.30E-05	−0.50544	0.99456
	AACT-3	c102447_g1	3.08	0.05	0	−0.40148	0.99421	−0.67435	0.99456
	HMGS	c61824_g1	4.19	0	0.71	−2.9209	0.35955	−3.6026	0.1887
	HMGR	c66823_g1	560.62	515.37	476.23	−0.58795	0.0030532	−0.48117	0.2103
	MK-1	c58223_g1	5.82	10.08	6.3	−0.20776	0.99421	0.43378	0.75911
	MK-2	c76083_g1	2.81	0	0	−3.3916	0.34457	−3.3936	0.27635
	PMK	c67242_g5	17.33	16.48	16.47	−0.42636	0.99421	−0.43222	0.99456
	MVD	c56352_g1	32.56	27.55	71.17	0.77398	0.00014198	−0.60355	0.99456
	IDI-1	c11491_g1	110.38	67.91	168.01	−1.2525	0.99421	−0.71196	0.99456
	IDI-2	c74391_g1	3.27	0	0	0.25038	0.0045152	−1.0658	0.0053014
MEP									
	DXS-1	c72095_g4	7.95	29.55	13.16	0.44095	0.27058	1.4152	1.02E-06
	DXS-2	c54473_g1	1.48	16.01	2.96	0.64894	0.99421	3.0798	8.03E-07
	DXR	c71143_g1	21.4	111.68	22.72	−0.26747	0.99421	2.0223	8.93E-24
	MCT	/	/	/	/				
	CMK	c61103_g1	7.83	20.07	7.43	−0.43182	0.99421	0.9928	0.072373
	MECPS	c61331_g1	24.64	102.4	32.58	0.045319	0.99421	1.6868	6.12E-10
	HDS	c71989_g1	12.68	81.59	15.17	−0.093038	0.99421	2.3319	4.61E-48
	HDR	c67492_g1	20.44	116.92	19.53	−0.41845	0.99421	2.1561	2.70E-30
IPP to Cholesterol									
	GPPS-1	c97556_g1	9.45	45.39	5.51	−1.1357	0.98019	1.8974	3.81E-06
	GPPS-2	c90599_g1	11.17	56.51	12.66	−0.17417	0.99421	1.9746	3.10E-09
	FPPS	c67887_g1	25.07	33.68	18.17	−0.81943	0.87666	0.061922	0.76188
	SS	c58461_g1	72.56	69.42	26.82	−1.7907	4.01E-06	−0.42756	0.99456
	SE	c56806_g1	247.1	11.46	48.8	−2.6935	6.27E-52	−4.7909	1.01E-90
	CAS	c73551_g1	66.58	244.71	39.38	−1.1029	0.00062765	−0.15504	0.70716
	SSR-1	c71749_g1	114.37	24.43	63.32	−1.2053	9.8524E-07	−2.5864	2.0831E-28
	SSR-2	c70423_g1	333.58	118.07	110.38	−1.9485	1.2352E-46	−1.8588	4.3029E-43
	SMO1	c62143_g1	787.76	15.24	131	−2.938	2.493E-172	−6.0534	2.5658E-285
	CPI	c57629_g1	16.77	11.25	42.91	1.0016	0.010324	−0.9308	0.92087
	CYP51	c56203_g1	1248.79	77.57	445.7	−1.8394	9.6484E-154	−4.3694	0
	C14-R	c69698_g1	62.54	21.13	43.66	−0.87364	0.26089	−1.9294	3.8533E-06
	8,7 SI	c69931_g1	57.39	36.66	70.93	−0.046848	0.48601	−1.0062	0.0167
	SMO2	c50487_g1	4.47	0	0.25	−4.5004	0.3795	NA	NA
	C5-SD	c67458_g1	580.81	245.51	146.88	−2.3389	8.7667E-70	−1.607	4.0749E-36
	7-DR	c60553_g1	74.97	111.84	64.56	−0.56945	0.94196	0.21529	0.0091335

Transcript abundance was calculated by Fragments Per kb per Million fragments (FPKM) values and only the unigenes at full length were subjected to this gene expression analysis. The “/” represents that the unigene was not at full length presented in the transcriptome.

## Supplementary Materials

The following are available online. Table S1: Annotation of all the *D. zingiberensis* unigenes based on public databases. Table S2: CD-HIT analysis of unigenes from *D. zingiberensis* transcriptome. Table S3: Blastp analysis of CYP amino acid sequences from *D. zingiberensis* with the CYP450 protein database from *Arabidopsis thaliana.* Table S4: Blastp analysis of UGT amino acid sequences from *D. zingiberensis* with the UGT protein database from *Arabidopsis thaliana.* Table S5: Annotation of all the putative cytochrome P450 unigenes from *D. zingiberensis* transcriptome. Table S6: Putative cytochrome P450 unigenes with the expression pattern in the order of FPKM(Aug_R) > FPKM(Nov_R) > FPKM(Aug_L). Table S7: Putative UDP-glycosyltransferase (UGT) unigenes with a length above 1000 bp from *D. zingiberensis* transcriptome. Figure S1: The UV absorption spectrum of the peak 2 in Figure 4.
